# Coding-complete genome of human alphaherpesvirus 1 isolated from a case of fulminant hepatitis

**DOI:** 10.1128/MRA.00355-23

**Published:** 2023-09-25

**Authors:** Natalie Viljoen, Felicity Burt, Jacqueline Weyer

**Affiliations:** 1 Center for Emerging Zoonotic and Parasitic Diseases, National Institute for Communicable Disease of the National Health Laboratory Service, Sandringham, South Africa; 2 Division of Virology, University of the Free State, Bloemfontein, South Africa; 3 Centre for Viral Zoonoses, Department of Medical Virology, University of Pretoria, Pretoria, South Africa; 4 Division of Virology, National Health Laboratory Service, Universitas, Bloemfontein, South Africa; 5 Department of Microbiology and Infectious Diseases, Faculty of Health Sciences, University of Witwatersrand, Johannesburg, South Africa; Portland State University, Portland, Oregon, USA

**Keywords:** hepatitis, human alphaherpes virus 1, herpesviruses

## Abstract

We report the coding-complete genome sequence of human alphaherpesvirus 1 (HHV1) isolated from a previously healthy 64-year-old male with fulminant hepatitis, a rare presentation of a common viral agent. The sequence is highly similar to previously described HHV1 sequences. Additional sequence data for fulminant hepatitis cases are required.

## ANNOUNCEMENT

Human alphaherpesvirus 1 (HHV1) belongs to the family *Herpesviridae* and is typically associated with mucocutaneous vesicular lesions, and disseminated disease is reported to be rare (1). HHV1 DNA was detected in the serum of a 64-year-old, previously healthy, male by qPCR confirming a diagnosis of fulminant herpetic hepatitis. Ethics approval was obtained from the Human Research Ethics Committee (Medical) at the University of the Witwatersrand (reference number: R14/49). Virus was isolated from serum (stored at −80°C) in Vero cells (ATCC CRL-1586), which were monitored for characteristic cytopathic effect (2). Cells were harvested after the second passage and concentrated from cleared supernatant using an Ultra-0.5 centrifugal filter with a 100-kDa cutoff (Amicon) in preparation for downstream processing for short- and long-read sequencing. For short-read sequencing, DNA was extracted using the Quick-DNA/RNA Viral Kit (Zymo Research), quantified using a Qubit fluorometer (Thermo Fisher Scientific), paired-end libraries prepared using the Nextera XT Library Prep kit (Illumina) for sequencing on the MiSeq (Illumina) platform (2 × 300 bp). For long-read sequencing, virus was purified through a 10% sucrose cushion, extracted using a genomic tip 20 /G (Qiagen), quantified using a Qubit fluorometer (Thermo Fisher Scientific), libraries prepared using the SMRTbell Express Template Prep kit 2.0 (Pacific BioSciences), DNA sheared using the Megaruptor 3 (Diagenode), and size selected using Ampure PB beads (Pacific BioSciences) for sequencing on the Sequel IIe system (Pacific BioSciences) using the Sequel II binding kit 2.2 and sequencing plate V2 (Pacific BioSciences).

FASTQ files were uploaded to the Galaxy Web platform and data analyzed using the server at http://usegalaxy.org (3). All tools were run using the default parameters, and short-read data were run for paired-end reads unless otherwise noted. Reads were quality trimmed (qualified quality phred, 20; length required for short reads, 40; length required for long reads, 100) using fastp V0.19.5 (4). For *de novo* assembly, reads were filtered against the reference sequence, assembled using MEGAHIT V1.2.9 (5) and evaluated using QUAST V5.2.0 (6). In addition, short and long reads were mapped on the reference sequence (GenBank accession number: NC001806.1 available at https://www.ncbi.nlm.nih.gov/nuccore/NC_001806) using Bowtie2 V2.4.5 (7) and Minimap2 V2.24 (8), respectively, and consensus sequence determined using iVar V1.3.1 (Minimum depth, 2) (9). Draft genomes obtained were aligned using MAFFT (10) and were in agreement. The most complete draft genome was obtained by long-read reference-based mapping, which was manually curated at ambiguous positions using Integrative Genomics Viewer V2.13.0 (11). The sequencing depth was determined using the SAMtools suite (12).

The genome is 152,063 bp in length (GC content 68.22%). Genome assembly metrics are provided in Table 1. The genome contains a 110 bp gap (position 117,349–117,458) in an inverted repeat region, which could not be resolved. Genome annotation was performed using NCBI ORF finder (13), DIAMOND (14), and the NCBI protein database (15). BLAST (16) analysis suggested that this sequence had a nucleotide identity of 98.75% with the reference sequence. Additional sequence data from HHV1 fulminant hepatitis cases are required to aid in studying potential virus-related factors that may play a role in the development of severe disease.

**TABLE 1:** Genome assembly metrics for short- and long-read data

	Short-read data (MiSeq, Illumina)	Long-read data (Sequel II, PacBio)
Total reads	2.8 million	14.1 thousand
Average read length	224 bp	3,675 bp
Genome coverage distribution for reference-based assembly	151,379 bp	152,159 bp
Average sequencing depth across genome	485×	12×
Genome coverage distribution with *de novo* assembly	134,520 bp	136,968 bp
Number of contigs for *de novo* assembly	11	9
N_50_ for *de novo* assembly	44,644 bp	45,163 bp

## Data Availability

This sequence has been deposited in GenBank under the accession number OQ102003.1 (https://www.ncbi.nlm.nih.gov/nuccore/OQ102003.1). The version described here is the first version. The raw reads were deposited in the NCBI Sequence Read Archive (SRA) under the accession number PRJNA913943 (https://www.ncbi.nlm.nih.gov/sra/SRX18774967 and https://www.ncbi.nlm.nih.gov/sra/SRX18774968).
